# Seroprevalence and risk factors of *Helicobacter pylori* infection among public sector employees in Kuwait

**DOI:** 10.1186/s13104-025-07598-1

**Published:** 2025-12-11

**Authors:** Hassan A. Bennakhi, Ahmad Al-Muhanna, Manaf A. Shehab, Jaber Al-Ali

**Affiliations:** 1 Dermatology Department, Russells Hall Hospital, The Dudley Group NHS Foundation Trust, Dudley, UK; 2 Surra Polyclinic, Surra, Kuwait; 3 Pediatrics Department, Ahmadi Hospital, Kuwait Oil Company, Ahmadi, Kuwait; 4 Faculty of Medicine, Kuwait University, Jabriya, Kuwait

**Keywords:** *Helicobacter pylori*, Prevalence, Risk factors, Public sector, Employees, Kuwait

## Abstract

**Objective:**

Approximately half of the global population is infected with *Helicobacter pylori*, which is associated with several diseases, including gastric adenocarcinoma. The prevalence of *H. pylori* infection among the general Kuwaiti population remains unknown. This cross-sectional study assessed the prevalence and associated risk factors of *H. pylori* infection among public sector employees in two governmental ministries in Kuwait.

**Results:**

Each participant underwent a diagnostic test for *H. pylori* IgG antibodies. A questionnaire was administered to collect data on demographic characteristics, lifestyle habits, and medical history for descriptive and inferential statistics, including logistic regression analysis. The cohort comprised 513 participants aged 20–72 years. Among them, 508 specified their sex, with 63.6% (323/508) being male. The prevalence of *H. pylori*-specific IgG antibodies, which indicates either a current or previous *H. pylori* infection, was 16.6% (85/513, 95% confidence interval [CI] 13%–20%). The > 50-year-old age group had the highest prevalence of *H. pylori* IgG antibody positivity. Male sex was the only independent factor significantly associated with a positive IgG result (adjusted odds ratio = 2.127, 95% CI 1.121–4.033, p-value = 0.021). A quantifiable percentage of the study population had IgG antibodies against *H. pylori*. Nevertheless, further studies with a larger, more representative sample size and more reliable *H. pylori* tests should be conducted to obtain more robust results.

**Supplementary Information:**

The online version contains supplementary material available at 10.1186/s13104-025-07598-1.

## Introduction

*Helicobacter pylori* is a short, helical, S-shaped, Gram-negative bacterium that mainly infects the pyloric region of the stomach [1]. It is associated with gastric adenocarcinoma, mucosa-associated lymphoid tissue lymphoma, chronic gastritis, and peptic ulcer disease [2]. Transmission occurs primarily via the fecal–oral routes and commonly spreads within households [3, 4]. *H. pylori* is the most common human bacterial pathogen, and it infects approximately half of the world’s population [4]. The key determinants of prevalence are childhood socioeconomic conditions, particularly hygiene, sanitation, and household crowding [4]. *H. pylori* is the highest known risk factor for gastric cancer [5], substantially impacting global health, and gastric cancer was ranked fifth in the World Health Organization’s GLOBOCAN 2022 report in terms of incidence and mortality [6]. Given its clinical and economic impacts, *H. pylori* remains a major global health burden.

### Prevalence of *H. pylori* in Kuwait

Current studies in Kuwait mainly focus on symptomatic patients. The prevalence of *H. pylori* infection among uninvestigated dyspeptic patients in Kuwait is 49.7% [7]. Further, only 7.3% of patients undergoing laparoscopic sleeve gastrectomy were infected [8]. Nevertheless, data on the prevalence of *H. pylori* infection in Kuwait are limited. To our knowledge, studies have not investigated the prevalence of *H. pylori* infection among the general population of Kuwait, nor have they looked at asymptomatic public sector employees.

### Rationale and significance

Determining the prevalence of *H. pylori* infection among Kuwaiti nationals provides baseline epidemiologic data and allows monitoring of trends over time, while identifying its associated risk factors can inform future prevention efforts. Although prevalence may change, the underlying risk factors are likely to remain relevant. During this study, 2.9% of Kuwaitis were unemployed, and 62.3% of employed Kuwaitis worked in the public sector in 2013 [9]. Therefore, a sample population of public sector employees would be ideal for assessing the prevalence of *H. pylori* infection among Kuwaiti nationals. Even though they represent a minority of the total population [10], Kuwaiti nationals remain the primary focus of government policies and lawmakers.

Although our data were collected over a decade ago, they remain highly relevant considering the relative socioeconomic stability of this population and the lack of major structural changes in public employment demographics over the last decade. Further, the scarcity of comparable epidemiological data from Kuwait increases the importance of these findings, particularly in the context of designing more representative and comprehensive national studies. Thus, we examined the results of a novel cross-sectional study conducted between 2013 and 2014. This research evaluated the prevalence of *H. pylori* infection among public sector employees in Kuwait and investigated its association with sociodemographic characteristics, behavioral aspects, and gastrointestinal symptoms.

## Materials and methods

### Study design and settings

This cross-sectional study was conducted among public sector employees in Kuwait. The minimum required sample size was estimated using the formula $\:S={Z}^{2}\times\:p\times\:(1-p)/{e}^{2}$. Assuming a population proportion ($\:p$) of 0.5, a 95% confidence level ($\:Z=1.96$), and a 5% margin of error ($\:e=0.05$), the calculated minimum sample size was 385. Data were collected from December 1, 2013, to October 10, 2014, at two governmental ministries: the Ministry of Justice and the Ministry of Finance, where employees participated voluntarily.

### Participants

**Fig. 1:**
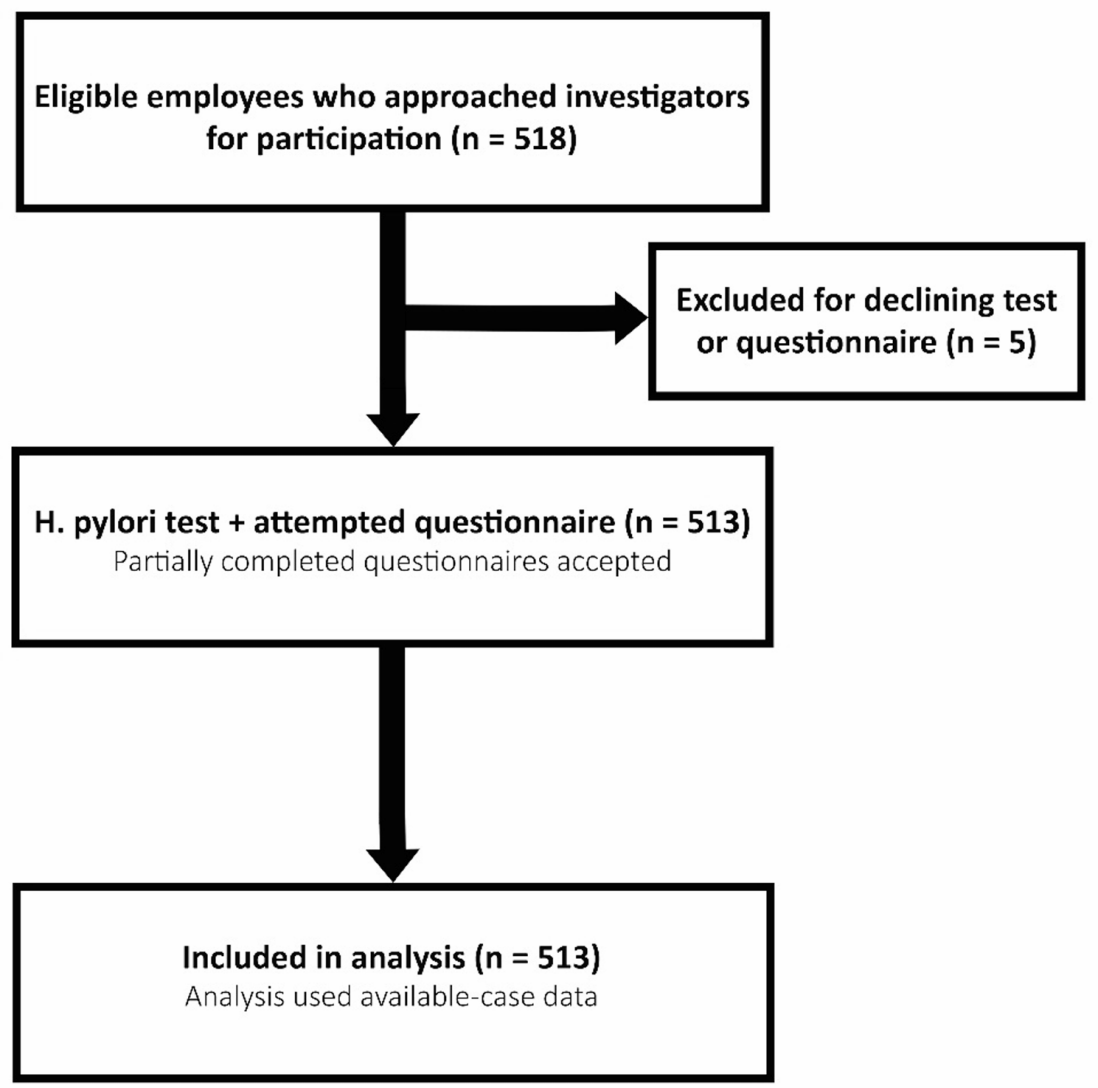
A participation flow diagram illustrating recruitment, exclusions, and the final analytical sample

The inclusion criteria were age ≥ 18 years, completion of the questionnaire, and *H. pylori* testing. Individuals who declined either the questionnaire or the test were excluded. Of 518 employees who participated, five were excluded, leaving 513 in the final sample. The STROBE flow diagram illustrates participant recruitment and inclusion (Fig. 1).

### Data collection procedures

Participants completed a structured paper-based questionnaire on sociodemographic characteristics, lifestyle factors, health history, and gastrointestinal symptoms. The questionnaire was developed specifically for this study, reviewed by a subject-matter expert, and provided in Arabic and English versions. Partially completed questionnaires were accepted. Considering the study’s descriptive design, no formal pilot testing or validation was performed. Supplementary File 1 shows the English language version of the questionnaire.

*H. pylori* IgG was assessed using a rapid chromatographic lateral-flow immunoassay kit (CLIA-05-9476, CLIA-waived Inc., San Diego, CA, USA). Capillary whole blood was collected via finger prick, and the kit’s reported sensitivity and specificity were 95.1% and 94.1%, respectively.

### Outcome measurements and variables

The primary outcome was *H. pylori* seropositivity, classified dichotomously (positive versus negative) based on the presence of both test and control lines on the rapid assay. Variables assesed included age, sex, nationality, marital status, education level, household crowding (persons per toilet), smoking status, hygiene practices, body mass index (BMI), and gastrointestinal symptoms.

### Ethical considerations

The current study was approved by Kuwait University’s Health Sciences Center (reference #304; 21/05/2013). Written informed consent was obtained from all participants before enrollment. Both ministries approved the study before recruitment. All study procedures followed the approved protocol by the Ethics Committee and the guidelines and regulations of Kuwait University and the relevant ministries. Data were anonymized.

### Statistical analysis

Results were analyzed using the Statistical Package for the Social Sciences (SPSS) software version 29.0.2.0 (IBM Corp., Armonk, NY, USA) for descriptive analyses and to measure associations (ORs and binary logistic regression), confidence interval (CIs), and significance. Pearson’s chi-squared test compared categorical groups, with two-sided p-values ≤ 0.05 considered statistically significant. Logistic regression analysis was performed to adjust for confounders, with the *H. pylori* test result as the dependent variable. The independent variables were those found to be statistically significant: sex, age, and nationality. The reference age group was the 20–30-year-old group, the reference sex was female, and the reference nationality was Kuwaiti. Missing data were not imputed. The Statistical Package for the SPSS was used to perform listwise deletion for multivariate analysis and pairwise deletion for descriptive and univariate analyses. Thus, only participants with available data for the variables of interest were included in each analysis.

## Results

### Demographic characteristics of the study cohort

Of the 518 ministerial employees who approached the study investigators, 5 who did not answer the questionnaire or undergo the *H. pylori* test were excluded (Fig. 1). Thus, the final cohort comprised 513 participants aged 20–72 (mean: 35.95 ± 9.84) years. Among them, 508 specified their sex, with 323 (63.6%) being male and 185 (36.4%) being female. There were 314 (62.2%) Kuwaiti nationals and 191 (37.8%) non-Kuwaiti nationals. The participants, including 124 (24.7%) from Al-Asimah (the capital governorate), 145 (28.8%) from Hawalli, 154 (30.6%) from Farwaniya, and 80 from distant governorates (Ahmadi, *n* = 30 [6.0%]; Jahra, *n* = 13 [2.6%]; and Mubarak Al-Kabeer, *n* = 37 [7.4%]), resided across all six Kuwaiti governorates.

### Prevalence of *H. pylori* infection and its associated factors

There were 85 (16.6%) study participants who tested positive for *H. pylori* (95% CI 13%–20%). The association between various factors—including demographic and socioeconomic characteristics (Table 1), behavioral patterns and hygiene practices (Table 2), and gastrointestinal symptoms—and a positive *H. pylori* test result was analyzed (Table 3). Several demographic factors, including male sex (odds ratio [OR]: 2.381, 95% CI 1.366–4.149, p-value = 0.002), age > 50 years (OR: 2.683, 95% CI 1.237–5.819, p-value = 0.013), and a non-Kuwaiti nationality (OR: 1.716, 95% CI 1.065–2.763, p-value = 0.026), were significantly associated with seropositivity (Table 1). None of the behavioral or hygiene-related factors, including smoking status, hand washing before handling food, bathing frequency, exercise frequency, BMI, and number of people sharing a toilet, were significantly associated with a positive *H. pylori* test result. The normal-weight participants had lower odds of infection than the other BMI groups. This association approached statistical significance in the overweight group (OR: 1.758, 95% CI 0.916–3.375, p-value = 0.090) (Table 2).

**Table 1:** Comparison between demographic and socioeconomic characteristics and their association with positive *H. pylori* test results

Characteristics of the participants	All participants	Participants with a positive H. pylori test	OR	95% CI		*p*-value^a^
	*N* (%)	*N* (%)		Lower	Upper	
Sex						
Female (R)	185 (36.4)	18 (9.7)				
Male	323 (63.6)	66 (20.4)	2.381	1.366	4.149	0.002
Age (years)						
20–30 (R)	190 (37.5)	21 (11.1)				
31–40	167 (33.0)	30 (18.0)	1.762	0.966	3.216	0.065
41–50	97 (19.2)	18 (18.6)	1.834	0.925	3.633	0.082
> 50	52 (10.3)	13 (25.0)	2.683	1.237	5.819	0.013
Marital status						
Single (R)	116 (22.8)	17 (14.7)				
Married	370 (72.8)	64 (17.3)	1.218	0.681	2.177	0.506
Divorced	22 (4.3)	3 (13.6)	0.920	0.245	3.449	0.901
Nationality						
Kuwaiti (R)	314 (62.2)	42 (13.4)				
Non-Kuwaiti	191 (37.8)	40 (20.9)	1.716	1.065	2.763	0.026
Governorate						
Capital (R)	124 (24.7)	18 (14.5)				
Hawalli	145 (28.8)	19 (13.1)	0.888	0.443	1.778	0.737
Farwaniya	154 (30.6)	36 (23.4)	1.797	0.963	3.352	0.066
Ahmadi (rural)	30 (6.0)	2 (6.7)	0.421	0.092	1.921	0.264
Jahra (rural)	13 (2.6)	2 (15.4)	1.071	0.219	5.236	0.933
Mubarak Al-Kabeer	37 (7.4)	5 (13.5)	0.920	0.317	2.674	0.878
Educational level						
Highschool or less (R)	89 (17.9)	18 (20.2)				
Intermediate diploma	175 (35.2)	29 (16.6)	0.783	0.408	1.505	0.464
University or above	233 (46.9)	35 (15)	0.697	0.371	1.309	0.262
Schooling						
Public (R)	451 (92.0)	71 (15.7)				
Private	39 (8.0)	8 (20.5)	1.381	0.610	3.128	0.439
Monthly family income (KD)						
< 500 (R)	119 (24.8)	25 (21.0)				
500 to < 1,000	111 (23.2)	20 (18.0)	0.826	0.429	1.591	0.568
1,000 to < 1,500	147 (30.7)	21 (14.3)	0.627	0.331	1.187	0.152
1,500 to < 2,000	39 (8.1)	3 (7.7)	0.313	0.089	1.102	0.071
> 2000	63 (13.2)	9 (14.3)	0.627	0.273	1.440	0.271

OR, odds ratio; CI, confidence interval; (R), reference; KD, Kuwaiti Dinars

^a^Pearson’s chi-squared test

**Table 2:** Comparison between behavioral and hygiene practices and their association with positive *H. pylori* test results

Characteristics of the participants	All participants	Participants with a positive H. pylori test	OR	95% CI		*p*-value^a^
	*N* (%)	*N* (%)		Lower	Upper	
Cigarette smoking status						
Non-smoker (R)	372 (74.3)	56 (15.1)				
Smoker	129 (25.7)	27 (20.9)	1.494	0.896	2.489	0.123
Hand washing before food handling						
Not always (R)	187 (37.9)	30 (16.0)				
Always	307 (62.1)	50 (16.3)	1.018	0.621	1.669	0.943
Bathing frequency						
Once weekly (R)	5 (1.0)	2 (40.0)				
Twice weekly	25 (5.0)	7 (28.0)	0.583	0.080	4.271	0.596
3–4 times weekly	147 (29.5)	19 (12.9)	0.223	0.035	1.420	0.112
Daily	322 (64.5)	54 (16.8)	0.302	0.049	1.852	0.196
Exercise frequency						
Never (R)	179 (35.7)	30 (16.8)				
Monthly or less	103 (20.5)	16 (15.5)	0.913	0.471	1.771	0.789
Weekly	138 (27.5)	19 (13.8)	0.793	0.425	1.479	0.466
Daily	82 (16.3)	17 (20.7)	1.299	0.670	2.520	0.439
People per toilet						
1 (R)	80 (16.4)	11 (13.8)				
2	196 (40.1)	30 (15.3)	1.134	0.538	2.390	0.742
3	94 (19.2)	18 (19.1)	1.486	0.656	3.366	0.343
≥ 4	119 (24.3)	21 (17.6)	1.344	0.609	2.967	0.464
Body mass index (kg/m^2^)						
Normal weight (18.5–24.9) (R)	136 (29.3)	15 (11.0)				
Underweight (< 18.5)	6 (1.3%)	1 (16.7)	1.613	0.176	14.752	0.672
Overweight (25.0–29.9)	190 (40.9)	34 (17.9)	1.758	0.916	3.375	0.090
Obese (≥ 30)	132 (28.4)	23 (17.4)	1.702	0.845	3.428	0.136

OR, odds ratio; CI, confidence interval; (R), reference

^a^Pearson’s chi-squared test

**Table 3:** Association of Gastrointestinal symptoms with positive *H. pylori* test results

Gastrointestinal symptoms	All patients	Patients with a positive H. pylori test	OR	95% CI		*p*-value^a^
	*N* (%)	*N* (%)		Lower	Upper	
Abdominal pain	240 (55.2)	39 (16.3)	0.887	0.537	1.464	0.639
Never (R)	195 (44.8)	35 (17.9)				
Rarely	152 (34.9)	24 (15.8)	0.857	0.485	1.514	0.595
Weekly	55 (12.6)	6 (10.9)	0.560	0.222	1.409	0.218
Daily	33 (7.6)	9 (27.3)	1.714	0.734	4.006	0.213
Heart burn	240 (55.8)	47 (19.6)	1.470	0.877	2.466	0.144
Never (R)	190 (44.2)	27 (14.2)				
Rarely	144 (33.5)	29 (20.1)	1.522	0.856	2.708	0.153
Weekly	60 (14.0)	10 (16.7)	1.207	0.547	2.665	0.641
Daily	36 (8.4)	8 (22.2)	1.725	0.712	4.179	0.227
Nausea	161 (38.7)	16 (9.9)	0.464	0.254	0.848	0.013
Never (R)	255 (61.3)	49 (19.2)				
Rarely	116 (27.9)	11 (9.5)	0.440	0.220	0.882	0.021
Weekly	37 (8.9)	4 (10.8)	0.510	0.172	1.506	0.223
Daily	8 (1.9)	1 (12.5)	0.601	0.072	4.995	0.637
Fullness after a small meal	207 (48.7)	35 (16.9)	1.064	0.637	1.777	0.813
Never (R)	218 (51.3)	35 (16.1)				
Rarely	114 (26.8)	18 (15.8)	0.980	0.527	1.822	0.950
Weekly	32 (7.5)	4 (12.5)	0.747	0.247	2.263	0.606
Daily	61 (14.4)	13 (21.3)	1.416	0.695	2.885	0.338
Belching	147 (37.3)	18 (12.2)	0.644	0.356	1.163	0.144
Never (R)	247 (62.7)	44 (17.8)				
Rarely	89 (22.6)	9 (10.1)	0.519	0.242	1.112	0.092
Weekly	25 (6.3)	1 (4.0)	0.192	0.025	1.459	0.111
Daily	33 (8.4)	8 (24.2)	1.476	0.625	3.490	0.375
Bloating	285 (63.8)	47 (16.5)	0.987	0.588	1.658	0.962
Never (R)	162 (36.2)	27 (16.7)				
Rarely	126 (28.2)	18 (14.3)	0.833	0.436	1.593	0.581
Weekly	70 (15.7)	13 (18.6)	1.140	0.549	2.367	0.725
Daily	89 (19.9)	16 (18.0)	1.096	0.555	2.165	0.792

OR, odds ratio; CI, confidence interval; (R), reference

^a^Pearson’s chi-squared test

Among the gastrointestinal symptoms, only nausea was significantly negatively associated with the test results (OR: 0.464, 95% CI: 0.254–0.848, p-value = 0.013). However, when nausea frequency was categorized as never, rarely, weekly, and daily, only rare occurrences were significantly negatively associated with a positive *H. pylori* test compared with never experiencing nausea (OR: 0.440, 95% CI: 0.220–0.882, p-value = 0.021). Further, weekly or daily nausea was not statistically significant, despite showing a negative association with a positive *H. pylori* test. The other gastrointestinal symptoms were positively associated with a positive *H. pylori* test when they occurred daily. However, none were statistically significant (Table 3).

Based on the multivariate analysis, male sex was the only independent predictor (adjusted OR: 2.127, 95% CI: 1.121–4.033, p-value = 0.021). Two age groups were approaching significance: the 31–40-year-old group (OR: 1.718, 95% CI: 0.930–3.173, p-value = 0.084) and the 41–50-year-old group (adjusted OR: 1.904, 95% CI: 0.948–3.824, p-value = 0.070). Age > 50 years, which showed a significant association with a positive *H. pylori* test in the initial analysis, did not show a significant association in the logistic regression analysis (OR: 1.814, 95% CI: 0.780–4.220, p-value = 0.167) (Table 4).

**Table 4:** Logistic regression analysis^a^ of variables identified as significant in the initial analysis

Characteristics of the participants	Adjusted OR	95% CI		*p*-value
		Lower	Upper	
Sex				
Female (R)	1.00			
Male	2.127	1.121	4.033	0.021
Age (years)				
20–30 (R)	1.00			
31–40	1.718	0.930	3.173	0.084
41–50	1.904	0.948	3.824	0.070
> 50	1.814	0.780	4.220	0.167
Nationality				
Kuwaiti (R)	1.00			
Non-Kuwaiti	1.033	0.586	1.821	0.910

OR, odds ratio; CI, confidence interval; (R), reference

^a^Binary logistic regression. Dependent variable: 0 = negative *H. pylori* test, 1 = positive *H. pylori* test. Independent variables: sex, age, and nationality

## Discussion

In the current study cohort, 16.6% of the participants tested positive for *H. pylori*, which is an important finding. However, these results should be interpreted with caution, as the sample may not represent the broader Kuwaiti population. First, this study exclusively focused on public sector employees, excluding other groups such as private sector employees, children, retirees, and the unemployed, thereby limiting its generalizability. In addition, our cohort comprised 62.2% of Kuwaiti nationals and 37.8% of non-Kuwaitis. This finding does not reflect the overall population in Kuwait in 2013, in which Kuwaiti nationals were the minority (33.6%) of the total population and non-Kuwaitis were the majority (66.4%) [9]. Further, as all participants were public sector employees, the cohort is more representative of the occupational distribution of Kuwaiti nationals as opposed to non-Kuwaitis because Kuwaiti nationals occupied 72.9% of public sector jobs in 2013 [9]. However, our study population offers insights into the overall *H. pylori* infection status among the employees of two of Kuwait’s government ministries. The male-to-female ratio is extremely similar to that of the overall Kuwaiti population, as the official estimates for 2013 reported that 55.6% of the total population were men. The proportion of ages represented in our sample is similar to the results of the 2011 national census, which reported that 74.3% of the total population was aged < 40 years [10]. This is reflected in our study cohort, in which 70.6% of participants were aged ≤ 40 years.

Compared with other countries, our results indicated a lower prevalence of *H. pylori* in Kuwait [11]. Several studies in West Asia and North Africa have reported high *H. pylori* prevalence infection among asymptomatic individuals. Rates include 71% in Nahavand, Iran [12], ; 64.2% among children in West Iran [13]; and 51% in Makkah, Saudi Arabia [14]. These studies showed that *H. pylori* prevalence increases with age. In Egypt, rates of 72.38% among schoolchildren [15] and 87.6% among individuals under 30 further indicate that infections commonly begin in early childhood [16]. However, a Saudi Arabian study reported a rate of 16.9% *H. pylori* seropositivity in those aged < 20 years and an overall prevalence of 28.8%, with higher rates in the older age groups [17]. Furthermore, they found that living in a rural area, crowded housing, and low socioeconomic status were independently associated with seropositivity. This may explain the slightly lower rate of seropositivity observed in our study, given the overall better quality of life, higher gross domestic product per capita, slightly younger population, higher level of urbanization, and lower unemployment rates in Kuwait [18, 19]. In our cohort, only 8.6% participants reported living in governorates that were considered rural, and the majority had either a university degree (46.9%) or intermediate education (35.2%).

Our prevalence aligns with reports from some European populations. A study in the Netherlands in 2013 reported a mean *H. pylori* seroprevalence for those born between 1977 and 1987 of 16% [20]. However, the aforementioned findings of a lower prevalence in some European countries are inconsistent. Another study performed in the Netherlands to determine *H. pylori* prevalence in pregnant women by measuring anti-*H. pylori* IgG and cytotoxin-associated gene product A antibodies found an overall seroprevalence of 46%, with Dutch women having a notably lower prevalence than non-Dutch women (24% vs. 64%) [21]. A study on *H. pylori* prevalence in Porto, Portugal, using an anti-*H. pylori* IgG enzyme-linked immunosorbent assay also found a high prevalence of 84.2% overall and 72.6% for those aged 18–30 years [22]. 

The prevalence of *H. pylori* in several Asian and African countries was also reported as high, reaching >50% in most countries [23]. Regional differences in the *H. pylori* prevalence likely reflect sanitation, hygiene, cultural practices, and socioeconomic conditions [24]. The lower prevalence observed in our study compared with regional and global estimates may be due to Kuwait’s higher living standards, widespread access to clean water and sanitation, robust public healthcare, lower household crowding, routine medical screening in government institutions, and accessible treatment through primary care. Improved childhood hygiene, including safer food preparation and reduced use of shared feeding utensils [25], may further reduce transmission and help explain lower prevalence in other Gulf countries.

In our study, male sex was the only independent factor significantly associated with a positive *H. pylori* test. Age > 50 years and non-Kuwaiti nationality were not statistically significant after adjustment in the logistic regression analysis, thereby indicating that their initial significance was attributed to confounding by male sex. This is supported by the demographic characteristics of our cohort, in which 95.3% (182/191) of non-Kuwaitis and 92.3% (48/52) of participants aged > 50 years were men. Consequently, male sex potentially confounded these associations.

Our findings are in contrast to those of other studies that did not find any association between *H. pylori* infection and sex [23]. Rather, socioeconomic factors such as low socioeconomic status, low education level, residence in rural areas or crowded homes, and consumption of contaminated water were found to be associated with *H. pylori* infection. However, our findings are in accordance with those of Replogle et al., who reported a significant association between male sex and *H. pylori* seropositivity, even after adjusting for confounders in a California-based study of 567 participants. This finding was further supported by a meta-analysis [26]. Replogle et al. showed that peptic ulcers and gastric cancers—both strongly associated with *H. pylori*—are 1.5–3 times more common in men, potentially because of a higher prevalence of *H. pylori* infection among the male participants. However, considering the inconsistency in existing data, further studies should be performed to confirm the correlation. Our univariate data analysis showed a negative association between some symptoms and *H. pylori* seropositivity, which should be interpreted cautiously. This may be because *H. pylori* infections are mostly asymptomatic [27, 28] or could reflect chance or reporting bias, as gastrointestinal symptoms were self-reported and subject to recall and perception variability.

This study uniquely explores the prevalence and risk factors of *H. pylori* infection among public sector employees in Kuwait, a population not previously examined. *H. pylori* infection is associated with numerous diseases, including gastric cancer. Hence, our findings should prompt further exploration of the health implications of the *H. pylori* prevalence found in our study. Further, they can be used as a baseline for future studies to determine whether the infection rate has increased or decreased since our study was performed. Literature from the Gulf region remains limited; therefore, our findings help address this gap and emphasize the need for larger, multicenter studies to validate the demographic, biological, and lifestyle determinants of *H. pylori* infection in Gulf states.

### Limitations

The current study had several limitations including the test method used, source of the sample population, selection method, and sample size. Considering the cross-sectional design, temporality or causality between identified factors and *H. pylori* infection could not be determined. Observed associations should be interpreted cautiously, as the selected test detects IgG antibodies, which cannot distinguish current infections from previous ones. The manufacturer claimed that the test had a sensitivity of 95.1% and a specificity of 94.1%. However, it would have been better to use a test that had undergone independent and rigorous scrutiny, given the relatively lower specificity of serologic assays [29, 30] and the availability of more accurate serology tests, such as enzyme immunoassay [31]. Consequently, the possibility of obtaining false-positive and/or false-negative results remains a concern. Participants from the public sector better represent Kuwaiti nationals, who are a minority population in Kuwait, while the majority of the population is non-Kuwaiti. Participants were recruited from two government ministries only (Finance and Justice), which may limit generalizability of the results. However, considering Kuwait’s centralized government structure, small geographic size, and employment distribution, this limitation is unlikely to have significantly affected the results. Another limitation is the selection method used because potential participants had to approach the study investigators to be recruited. This introduced self-selection bias and might have had an effect on the results (i.e., those who had undergone recent testing for *H. pylori* infection or those who knew that they were infected and might have decided not to approach the study investigators). Further, data collection via self-reported questionnaires might have introduced recall or social desirability bias, particularly in responses concerning hygiene practices and symptoms. The sample size could have affected the association analysis, and a larger sample size might have revealed stronger associations. As job role data were not collected, the *H. pylori* tests between junior and senior level employees could not be compared, and this is another limitation of this study.

## Supplementary Information

Below is the link to the electronic supplementary material.

Supplementary Material 1

## Data Availability

The datasets generated during and/or analyzed during the current study are available from the corresponding author upon reasonable request.

## Abbreviations

BMI: Body mass index
CA, USA: California, United States of America
CI: Confidence interval
GCC: Gulf Cooperation Council
GLOBOCAN: Global Cancer Observatory
H. pylori: Helicobacter pylori
IgG: Immunoglobulin G (antibody)
KD: Kuwaiti Dinars
NY, USA: New York, United States of America
OR: Odds ratio
p-value: Probability value
R: Reference category
SPSS: Statistical Package for the Social Sciences
STROBE: Strengthening the Reporting of Observational Studies in Epidemiology
UK: United Kingdom
WHO: World Health Organization
