# Supplementary material for: Seroprevalence and risk factors of Helicobacter pylori infection among public sector employees in Kuwait
Source: BMC Res Notes. 2025 Dec 11;19:21. doi: 10.1186/s13104-025-07598-1 (PMC12801954; doi:10.1186/s13104-025-07598-1)
Supplement: Supplementary file 1 — Supplementary Material 1 [file 13104_2025_7598_MOESM1_ESM.docx]

**
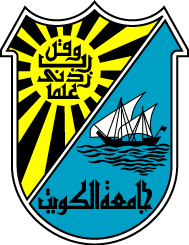

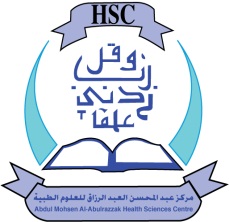
**

Serial number

Department of Medicine

Faculty of Medicine

Kuwait University

**Prevalence and Associated Factors of Stomach Bacteria**

**(Helicobacter Pylori) Infection Among the Adult Population in Kuwait**

Please tick (✓) the box that you choose or write your response in the space provided:

**Section I: Sociodemographic Characteristics**

| 1. What is your gender? | | | | | | | | | |  | | ^1^ Male | | | | | | | | |  | | ^2^ Female | | | | | | | | | | | | | | | | | | | |
| --- | --- | --- | --- | --- | --- | --- | --- | --- | --- | --- | --- | --- | --- | --- | --- | --- | --- | --- | --- | --- | --- | --- | --- | --- | --- | --- | --- | --- | --- | --- | --- | --- | --- | --- | --- | --- | --- | --- | --- | --- | --- | --- |
|  | | | | | | | | | | | | | | | | | | | | | | | | | | | | | | | | | | | | | | | | | | |
| 2. What is your age? | | | | | | | |  |  | | years | | | | | | | | | | | | | | | | | | | | | | | | | | | | | | | |
|  | | | | | | | | | | | | | | | | | | | | | | | | | | | | | | | | | | | | | | | | | | |
| 3. What is your marital status? | | | | | | | | | | | | |  | | ^1^ Single | | | | | | | | | |  | | | ^2^ Married | | | | | |  | | | ^3^ Divorced | | | | | |
|  | | | | | | | | | | | | | | | | | | | | | | | | | | | | | | | | | | | | | | | | | | |
| 4. What is your nationality? | | | | | | | | | | | | | | | | | | | | | | | | | | | | | | | | | | | | | | | | | | |
|  | |  | ^1^ Kuwaiti | | | | | | | | | | | |  | | | ^2^ Non-Kuwaiti (Please specify:___________________) | | | | | | | | | | | | | | | | | | | | | | | | |
|  | | | | | | | | | | | | | | | | | | | | | | | | | | | | | | | | | | | | | | | | | | |
| 5. In which governorate do you live? | | | | | | | | | | | | | | | | | | | | | | | | | | | | | | | | | | | | | | | | | | |
|  | ^1^ Capital | | | | | | | | | | | | | | |  | | | ^2^ Hawalli | | | | | | | | | | | | | | | | |  | | | ^3^ Farwania | | | |
|  | | | | | | | | | | | | | | | | | | | | | | | | | | | | | | | | | | | | | | | | | | |
|  | ^4^ Al-Ahmadi | | | | | | | | | | | | | | |  | | | ^5^ Al-Jahra | | | | | | | | | | | | | | | | |  | | | ^6^ Mubarak Al-Kabeer | | | |
|  | | | | | | | | | | | | | | | | | | | | | | | | | | | | | | | | | | | | | | | | | | |
| 6. What is your highest level of education? | | | | | | | | | | | | | | | | | | | | | | | | | | | | | | | | | | | | | | | | | | |
|  | ^1^ High school or less | | | | | | | | | | | | |  | | | ^2^ Intermediate diploma | | | | | | | | | | | | | | | | | | | |  | | | | ^3^ University or above | |
|  | | | | | | | | | | | | | | | | | | | | | | | | | | | | | | | | | | | | | | | | | | |
| 7. What is your father's highest level of education? | | | | | | | | | | | | | | | | | | | | | | | | | | | | | | | | | | | | | | | | | | |
|  | ^1^ High school or less | | | | | | | | | | | | |  | | | ^2^ Intermediate diploma | | | | | | | | | | | | | | | | | | | |  | | | | ^3^ University or above | |
|  | | | | | | | | | | | | | | | | | | | | | | | | | | | | | | | | | | | | | | | | | | |
| 8. What is your mother's highest level of education? | | | | | | | | | | | | | | | | | | | | | | | | | | | | | | | | | | | | | | | | | | |
|  | ^1^ High school or less | | | | | | | | | | | | |  | | | ^2^ Intermediate diploma | | | | | | | | | | | | | | | | | | | |  | | | | ^3^ University or above | |
|  | | | | | | | | | | | | | | | | | | | | | | | | | | | | | | | | | | | | | | | | | | |
| 9. What is your family's monthly income in Kuwaiti Dinars? | | | | | | | | | | | | | | | | | | | | | | | | | | | | | | | | | | | | | | | | | | |
|  | ^1^ Less than 500 | | | | | | | | | | | |  | | ^2^ 500 to less than 1000 | | | | | | | | | | | | | | | | | | | |  | | | ^3^ 1000 to less than 1500 | | | | |
|  | | | | | | | | | | | | | | | | | | | | | | | | | | | | | | | | | | | | | | | | | | |
|  | | | |  | | ^4^ 1500 to less than 2000 | | | | | | | | | | | | | | | | | | | | | | | | | |  | ^5^ 2000 and more | | | | | | | | | |
|  | | | | | | | | | | | | | | | | | | | | | | | | | | | | | | | | | | | | | | | | | | |
| 10. How many bedrooms are there in your house? | | | | | | | | | | | | | | | | | | | | | | | | | |  | | |  | | rooms | | | | | | | | | | | |
|  | | | | | | | | | | | | | | | | | | | | | | | | | | | | | | | | | | | | | | | | | | |
| 11. How many persons are living in your house? | | | | | | | | | | | | | | | | | | | | | | | | | | |  | | |  | | persons | | | | | | | | | | |
|  | | | | | | | | | | | | | | | | | | | | | | | | | | | | | | | | | | | | | | | | | | |
| 12. How many persons share a toilet in your house? | | | | | | | | | | | | | | | | | | | | | | | | | | | | | | | | | | | | | | | | | | |
|  | ^0^ None | | | |  | | ^1^ Two persons | | | | | | | | | | | | | | |  | | ^2^ Three persons | | | | | | | | | | | | | | | |  | | ^3^ Four and more |
| 13. What type of school did you go to? | | | | | | | | | | | | | | | | | | | | | | | | | | | | | | | | | | | | | | | | | | |
|  | |  | ^1^ Public school | | | | | | | | | | | | | | | | | | | |  | | ^2^ Private school | | | | | | | | | | | | | | | | | |
|  | | | | | | | | | | | | | | | | | | | | | | | | | | | | | | | | | | | | | | | | | | |
| 14. What is your weight? | | | | | | | | | |  | | |  | | | |  | | | kg | | | | | | | | | | | | | | | | | | | | | | |
|  | | | | | | | | | | | | | | | | | | | | | | | | | | | | | | | | | | | | | | | | | | |
| 15. What is your height? | | | | | | | | | |  | | |  | | | |  | | | cm | | | | | | | | | | | | | | | | | | | | | | |

**Section II: Behavioral Aspects**

| 16. Do you smoke cigarettes? | | | | | |  | ^0^ No | | |  | ^1^ Yes | | | | | | | | |
| --- | --- | --- | --- | --- | --- | --- | --- | --- | --- | --- | --- | --- | --- | --- | --- | --- | --- | --- | --- |
|  | | | | | | | | | | | | | | | | | | | |
| 17. Do you wash your hands before handling food? | | | | | | | | | | | |  | ^0^ Not Always | | | |  | | ^1^ Always |
|  | | | | | | | | | | | | | | | | | | | |
| 18. How often do you take a bath? | | | | | | | | | | | | | | | | | | | |
|  | ^1^ Once a week |  | | ^2^ Twice a week | | | |  | ^3^ 3-4 times a week | | | | | | |  | | ^4^ Daily | |
|  | | | | | | | | | | | | | | | | | | | |
| 19. How often do you exercise? | | | | | | | | | | | | | | | | | | | |
|  | ^0^ Never | |  | | ^1^ Daily | | |  | ^2^ Weekly | | | | |  | ^3^ Monthly or less | | | | |

**Section III: Health and Co-Morbidities**

| 20. Have you been diagnosed by a doctor with any of the following conditions? *(you may choose more than one)* | | | | | | | | | | | | | | | | | | | | |
| --- | --- | --- | --- | --- | --- | --- | --- | --- | --- | --- | --- | --- | --- | --- | --- | --- | --- | --- | --- | --- |
|  | | | | | | | | | | | | | | | | | | | | |
|  | | | | | | | | | | | | | | | | | | | | |
| **Condition** | | | | | | **No** | | | | | | | **Yes** | | | | | | | |
|  | | | | | | | | | | | | | | | | | | | | |
|  | | | | | | | | | | | | | | | | | | | | |
| a. Diabetes | | | | | | | |  | ^0^ | | | | | | | |  | ^1^ | | |
|  | | | | | | | | | | | | | | | | | | | | |
| b. Hypertension | | | | | | | |  | ^0^ | | | | | | | |  | ^1^ | | |
|  | | | | | | | | | | | | | | | | | | | | |
| c. High cholesterol levels | | | | | | | |  | ^0^ | | | | | | | |  | ^1^ | | |
|  | | | | | | | | | | | | | | | | | | | | |
| d. Asthma | | | | | | | |  | ^0^ | | | | | | | |  | ^1^ | | |
|  | | | | | | | | | | | | | | | | | | | | |
| e. Stomach ulcer | | | | | | | |  | ^0^ | | | | | | | |  | ^1^ | | |
|  | | | | | | | | | | | | | | | | | | | | |
| f. Stomach cancer | | | | | | | |  | ^0^ | | | | | | | |  | ^1^ | | |
|  | | | | | | | | | | | | | | | | | | | | |
| Please write down the conditions that you have received treatment for:_____________________ | | | | | | | | | | | | | | | | | | | | |
| 21. Do you have any of the following symptoms? *( you may choose more than one)* | | | | | | | | | | | | | | | | | | | | |
|  | | | | | | | | | | | | | | | | | | | | |
|  | | | | | | | | | | | | | | | | | | | | |
| **Symptom** | **Never** | | | | | | **Rarely** | | | | **Weekly** | | | | | **Daily** | | | | |
|  | | | | | | | | | | | | | | | | | | | | |
|  | | | | | | | | | | | | | | | | | | | | |
| a. Abdominal pain | | | |  | ^0^ | | |  | ^1^ | | | | |  | ^2^ | | | |  | ^3^ |
|  | | | |  |  | | |  |  | | | | |  |  | | | |  |  |
| b. Heart burn | | | |  | ^0^ | | |  | ^1^ | | | | |  | ^2^ | | | |  | ^3^ |
|  | | | |  |  | | |  |  | | | | |  |  | | | |  |  |
| c. Nausea | | | |  | ^0^ | | |  | ^1^ | | | | |  | ^2^ | | | |  | ^3^ |
|  | | | |  |  | | |  |  | | | | |  |  | | | |  |  |
| d. Full after a small meal | | | |  | ^0^ | | |  | ^1^ | | | | |  | ^2^ | | | |  | ^3^ |
|  | | | |  |  | | |  |  | | | | |  |  | | | |  |  |
| e. Belching | | | |  | ^0^ | | |  | ^1^ | | | | |  | ^2^ | | | |  | ^3^ |
|  | | | |  |  | | |  |  | | | | |  |  | | | |  |  |
| f. Bloating | | | |  | ^0^ | | |  | ^1^ | | | | |  | ^2^ | | | |  | ^3^ |
|  | | | |  |  | | |  |  | | | | |  |  | | | |  |  |
|  | | | |  |  | | |  |  | | | | |  |  | | | |  |  |
|  | | | | | | | | | | | | | | | | | | | | |
| 22. Have you been treated for the stomach bacteria (Helicobacter Pylori) infection before? (The treatment consists of two antibiotics and a medication that reduces stomach acidity for one to two weeks) | | | | | | | | | | | | | | | | | | | | |
|  | |  | ^0^ No | | | | | | |  | | ^1^ Yes | | | | | | | | |
|  | | | | | | | | | | | | | | | | | | | | |
| 23. Have you taken antibiotics and pills that reduce gastric acidity simultaneously for a week or more in the past? | | | | | | | | | | | | | | | | | | | | |
|  | |  | ^0^ No | | | | | | |  | | ^1^ Yes | | | | | | | | |

- THANK YOU FOR YOUR COOPERATION -
